# Hepatitis viruses in Kathmandu, Nepal: hospital-based study

**DOI:** 10.1186/s13104-018-3739-1

**Published:** 2018-08-30

**Authors:** Birendra Prasad Gupta, Anurag Adhikari, Santosh Chaudhary

**Affiliations:** 1 Central Diagnostic Laboratory and Research Centre, Kathmandu, Nepal; 2 Kathmandu Research Institute for Biological Science, Kathmandu, Nepal; 3 MB Kedia Dental College Private Limited, Birgunj, Nepal

**Keywords:** Hepatitis virus, Prevalence, Kathmandu, Nepal

## Abstract

**Objective:**

The objective of this study was to see the aetiology and outcome of sporadic acute viral hepatitis (AVH) in Kathmandu, Nepal.

**Results:**

Among 210 patients, 94 (45%) were male and 116 (55%) were female. Mean age was 30 years. 52 (24.7%) out of 210 were positive for either of the hepatitis virus infection. Major causative agent for AVH among hepatitis positive patients were hepatitis E virus (HEV) in 36 (69.2%), followed by hepatitis A virus (HAV) 8 (15.3%), hepatitis B virus (HBV) 7 (13.4%) and hepatitis C virus (HCV) 1 (1.9%). The 158 (75.3%) patient were negative for all hepatitis viral markers. Co-infections with more than one virus were found in 4 (7.6%) patients. All liver-specific enzymes including bilirubin increased in hepatitis-infected patients. We found large number circulation of HEV in Kathmandu, Nepal, indicating that this region is endemic for hepatitis virus infection.

## Introduction

Liver disease due to hepatitis virus is a major public health problem in the world affecting millions of people worldwide [1]. A unique group of viruses hepatitis A (HAV), hepatitis B (HBV), Hepatitis C (HCV), and hepatitis E (HEV) are responsible for the liver disease. All these viruses are associated with significant morbidity and mortality in developing and developed countries [2]. In 2015, WHO estimates 1.34 million deaths due to viral hepatitis where HBV, HCV is a major causative agent for the death [3]. The clinical outcome of viral hepatitis can range from subclinical to life-threatening infection [2, 4, 5]. The HAV and HEV are transmitted through ingestion of contaminated food or water infected fecal material [6]; whereas, HBV and HCV are transmitted primarily via the perinatal route, involving salivary exchange, sexual contact, vertical transmission from mother to offspring, and exposure to infected blood products [7].

HAV and HEV mostly affects young children and young adults respectively and are endemic in many developing countries in Asia and Africa [8]; whereas HBV and HCV infection are mainly reported from adults and found sporadically in Western Europe, North America, and other developed countries [9]. Most of the HAV and HEV cases are self-limiting and clinically undetectable whereas 70–80% of people who are acutely infected with HBV and HCV progress to chronic hepatitis [10, 11].

Until recently, there have been few reliable (sensitive and specific) commercial assays for detecting antibodies to hepatitis viruses (HAV, HEV, HCV, and HBV) which can be used for routine diagnosis. Although, recently, newer assays have been developed that show high sensitivity and specificity, allowing more accurate detection/diagnosis of hospital cases. However, the high cost of these assays has a limited assessment in developing countries like Nepal. The detailed information regarding the present situation of infections due to hepatitis viruses in Nepal is still insufficient to understand hepatitis disease burden. The aim of this study was to determine the prevalence of hepatitis viruses (HAV, HBV, HCV, and HEV) in jaundice patients at a tertiary hospital in Kathmandu.

## Main text

### Methods

#### Study design and serological assay

Clinical information and blood samples were collected from the patients suffering from jaundice at the pathology department, Capital Hospital, Kathmandu, Nepal from April 2014–December 2014. The liver function test was carried out and remaining serum was subjected for the detection of IgM antibodies against HAV, HCV, and HEV using commercial kits produced by Wantai (Beijing, China) according to manufacturer’s instructions. Similarly, HBsAg was assessed using commercially available enzyme immunoassays Enzygnost, Dade Behring, Germany.

#### HEV RNA detection by real-time PCR

RNA was extracted from HEV IgM positive sample for further confirmation using quantitative reverse transcript PCR. HEV RNA was isolated from 140 μl of serum sample using Nucleospin viral RNA isolation kit (MACHEREY–NAGEL, Germany) according to the manufacturer’s instruction [12]. The standard protocol for HEV RNA assay was adapted from a previously described study by Jothikumar et al. [13]. The 25 μL reaction mixture contained 12.5 μL of 2× One-Step RT-PCR reaction buffer (Genmagbio, Beijing, China), 0.2 μL of GenMagScript RT Enzyme Mix, 5 μL of RNA, and primers (forward primer: BDHEVF (5′-GGTGGTT TCTGGGGTGAC-3′) and reverse primer: BDHEVR (5′-A GGGGTTGGTTGGATGAA-3′) and probe (BDHEVP; 5′-TGATTCTCAGCCCTTCGC-3′) at concentrations of 200 nM [14]. Additionally, internal controls, supplied in the kit (GenMagScript, China), were used to identify possible PCR inhibition as detected by fluorescence channel orange in Rotor-gene Q. As an exogenous internal control sequence, lambda gene PCR product (278-bp) was added to the reaction mixture [14].

#### Statistical analyses

The statistical analyses were performed using SPSS version 11.0 software (SPSS Inc., Chicago, IL, USA). Data are presented as mean ± SD and differences were considered statistically significant at two-sided p-values ≤ 0.05.

### Results

A total number of 52 (24.7%) out of 210 jaundice cases were found positive for hepatitis virus positive (Table 1). The prevalence rate in female (55.8%) was higher than the male (44.2%). Mean age of hepatitis virus positive was 30 years, where 13 years old young male adult were positive for HCV (Table 2). Major causative agent for acute viral hepatitis (AVH) among hepatitis positive patients were hepatitis E virus (HEV) in 36 (69.2%), followed by hepatitis A virus (HAV) 8 (15.3%), hepatitis B virus (HBV) 7 (13.4%) and hepatitis C virus (HCV) 1 (1.9%). The 158 (75.3%) patient were negative for all hepatitis viral markers. Co-infections with more than one virus were found in 4 (7.6%) patients. Comparing the positive rate of anti-HEV IgM, HBsAg, anti-HAV IgM and anti-HCV Ab, the data significantly implicated hepatitis E virus as the main etiological agent of the clinical symptoms of jaundice. All liver-specific enzymes including bilirubin were increased in hepatitis-infected patients (Table 3). We also observed a low prevalence for HEV RNA 12 (33.3%) out 36 of among the hepatitis E positive cases suggesting low viremia of HEV during a hospital visit. Moreover, we observed a marked increase in hepatitis seropositive cases during the rainy season (May to August) of Nepal.

**Table 1:** Demographic details of Jaundice patients visited in the hospital during the study period

Characteristic	Hepatitis virus positive (%) [*n* = 52]	Hepatitis virus negative (%) [*n* = 158]
Age (years)		
< 20	5 (9.6)	21 (13.3)
21–40	35 (67.3)	97 (61.4)
41–59	7 (13.5)	28 (17.7)
> 60	5 (9.6)	12 (7.6)
Mean age	27	35
Gender		
Male	23 (44.2)	69 (39)
Female	29 (55.8)	97 (61)

**Table 2:** Gender wise distribution of hepatitis virus-positive patients

	Hepatitis virus positive				
	HAV	HBV	HCV	HEV	Total
Gender					
Male	2 (3.8)	5 (9.6)	1 (1.9)	15 (28.8)	23 (44.2)
Female	6 (11.5)	2 (3.8)	0 (0.0)	21 (40.3)	29 (55.8)
Total	8 (15.3)	7 (13.4)	1 (1.9)	36 (69.2)	52 (100)
Mean age (years)	31	33	13	31

**Table 3:** Biochemical parameters in hepatitis positive patients

Biochemical parameters	HAV	HBV	HCV	HEV
Total bilirubin (0.2–1.3 mg/dL)	2.2	1.3	0.7	1.8
Direct bilirubin (< 0.3 mg/dL	1.3	0.4	0.2	0.8
ALT (13–69 IU/L)	38.3	38.8	30	118.2
AST (15–46 IU/L)	49.8	40.4	24	100.4
ALP (38–109 IU/L)	126.6	105.8	23	164.6

### Discussion

Sporadic and the outbreak of hepatitis viruses (A–E) has been reported from developing as well as developed countries every year with 1.34 million death in 2015, which is higher than tuberculosis and HIV [3]. As compared with past decades; mortality due to hepatitis viruses are increasing these days, this might be, because of in past there were only HAV and HBV, however; HCV, HDV, and HEV are more common. The present data indicate a high prevalence of HEV in Nepal where the massive outbreak occurred in the Kathmandu Valley in 1973 and 1981–1982, 1985 with the recent outbreak in Biratnagar where more than 6000 individuals were affected with 17 reported deaths in 2014 [15–18]. During the outbreak, most of the cases were young adults, and pregnant women especially experienced acute liver failure and mortality.

In Nepal, hepatitis A virus infection was most common in children till last decades, it is emerging as an important cause of acute hepatitis in young adults [19]. Prevalence of HBV 1.1% in overall population, however; some ethnic groups in the Himalayan region have high incidence rates [20]. HBV is one of the major causative agents for liver cirrhosis and hepatocellular carcinoma; although the prevalence rate is low in the general population in Nepal. Similarly, the overall prevalence of HCV is only 0.4% of the general population in the country, however, it was found up to 19% in HIV infected individuals [21]. There is a number of hospital-based reports, sporadic cases and outbreak due to HAV, and HEV in Nepal; however limited scientific publication available online [20, 22–24].

Although hospital-based data presented in this study will not represent the entire country, the disease burden due to these viral infections seem to be alarming these days and concern authorities should priorities to overcome with viral hepatitis in the country.

Both HEV and HAV infections which are mostly related to poor hygiene and are spread by the fecal–oral route are reported as outbreak from Kathmandu in the past with recent outbreak in Biratnagar in 2014 are vaccine preventable. Although HAV vaccine is available in the national immunization programme; Hecolin-239 vaccine for HEV is not available outside China because it has not been pre-qualified by WHO. One interesting issue is the very high prevalence of HEV in the Kathmandu valley where female were more infected than male. This demonstrates that increase chances of mortality in pregnant women. Our study has the similar finding with the Gupta et al. and Shrestha et al. and his colleague in relation to the prevalence of those two viruses in Nepal [8, 20]. In their study, they found that HEV is more common than HAV in the Kathmandu valley. The higher prevalence of antibody against HEV in Kathmandu where most of the outbreak occurred in the past [14]. It is not clear why the outbreak of HEV mostly occurred in Kathmandu valley. Improved food and water sanitation probably reduce the number of the outbreak in the future. The hospital and community based systematic study of at least HAV and HEV in the different geographical region (Himalayan, Hilly and Terai region) can reveal the facts. We found high prevalence of HEV infection during the rainy season which might be associated with the fecal contamination of the water supply because ongoing road and Melamchi Water Supply Project constitution for a decade and 78.7% coliform is reported from common drinking water from jar in the year 2018 which threatens the life of many people in this country (National Kantipur news report).

HBV cases are still reported from different hospital of Nepal although hepatitis B vaccine has been available for about 20 years in the country, there are still many people in the rural area in Nepal who do not benefit from its protection, this might be due to its high cost and less priority for the policymaker at Government level. We also observed a high prevalence of HBsAg in jaundice cases in our study than the government annual report because only 0.9% of the population was reported to be positive for HBsAg [20]. Seroprevalence of HBV infection is not uniform and varies in a different region and different ethnic groups in the country [25].

Seroprevalence of HCV is only 0.4% of the general population, however; high seroprevalence was found among intravenous drug user and HIV infected individuals. Although, exact reasons for high prevalence in these population remains unclear; blood transfusion without proper screening, use of unsterile injections might be one of the factors for the spread of HCV in this country. The detailed study needs to be carried out in the future because HCV is the one of the major cause of liver cirrhosis and hepatocellular carcinoma in chronic patients.

### Conclusion

Changing epidemiology and dynamics of hepatitis viruses (A–E) in Nepal alarming and detailed study should be carried out to understand the actual disease burden caused by these viruses. In a hospital-based study, we observed a high prevalence of HEV, HAV and HBV in the Nepalese population with jaundice and elevated liver enzymes. We found HEV is a major causative agent for Jaundice followed by HAV, HBV, and HCV indicating that group of hepatitis virus to cause a serious health problem in this country with a large population. Detailed studies including well designed systematic surveillance with a randomized selection of persons probably give us more precise information on the overall prevalence rate of those viral infections in this beautiful Himalayan country. We believe urgent standard public health approaches implementation like provision of clean water, proper disposal of sewage and improved personal hygiene through health education and implementation of HEV vaccine should be taken which might help control HEV infections in the future. Similarly, mass vaccination with HBV should be initiated and it should reach to a rural village in Nepal to prevent HBV infection in the future because HBV vaccination has not been achieved by Government of Nepal as expected. This might be the unaware of this vaccine or financial region because of the high cost of the vaccine. In addition, regular quality control of kit used in blood bank will help to prevent transfusion of HBV infected blood to the recipient. Since the vaccine is not available for HCV, an increasing awareness in dealing with blood and blood products might help to decrease the spread of HCV infection in this country. In conclusion, an available vaccine for HAV and HBV should reach to each people and license of HEV vaccine should be initiated to overcome with HEV outbreak and prevent the loss of life from HEV in pregnant women.

## Limitation

The author could not perform sequencing of HEV isolate because of a limited budget.

## Abbreviations

ALP: alkaline phosphatase
ALT: alanine aminotransferase
AST: aspartate aminotransferase
HAV: hepatitis A virus
HBV: hepatitis B Virus
HBsAg: hepatitis B surface antigen
HCV: hepatitis C Virus
HEV: hepatitis E virus
HIV: human immunodeficiency virus
PCR: polymerase chain reaction
WHO: World Health Organization
